# 2-Amino-5-bromo­pyridinium 3-carb­oxy-4-hy­droxy­benzene­sulfonate

**DOI:** 10.1107/S1600536810033908

**Published:** 2010-08-28

**Authors:** Madhukar Hemamalini, Hoong-Kun Fun

**Affiliations:** a X-ray Crystallography Unit, School of Physics, Universiti Sains Malaysia, 11800 USM, Penang, Malaysia

## Abstract

The asymmetric unit of the title salt, C_5_H_6_BrN_2_^+^·C_7_H_5_O_6_S^−^, contains two independent 2-amino-5-bromo­pyridinium cations and two independent 3-carb­oxy-4-hy­droxy­benzene­sulfonate anions. The hy­droxy and carboxyl groups of the anions are involved in intra­molecular O—H⋯O hydrogen bonds, which generate *S*(6) ring motifs. In the crystal structure, the ions are linked by N—H⋯O and O—H⋯O hydrogen bonds into a two-dimensional network parallel to (110). Adjacent networks are linked *via* C—H⋯O hydrogen bonds.

## Related literature

For applications of pyridinium compounds, see: Akkurt *et al.* (2005 ▶); Navarro Ranninger *et al.* (1985 ▶); Krizanovic *et al.* (1993 ▶); Luque *et al.* (1997 ▶); Qin *et al.* (1999 ▶); Yip *et al.* (1999 ▶); Lah *et al.* (2002 ▶); Ren *et al.* (2002 ▶); Rivas *et al.* (2003 ▶); Luque *et al.* (1997 ▶); Jin *et al.* (2000 ▶); Albrecht *et al.* (2003 ▶). For related structures, see: Hemamalini & Fun (2010 ▶); Quah *et al.* (2010 ▶). For hydrogen-bond motifs, see: Bernstein *et al.* (1995 ▶). For the stability of the temperature controller used in the data collection, see: Cosier & Glazer (1986 ▶).
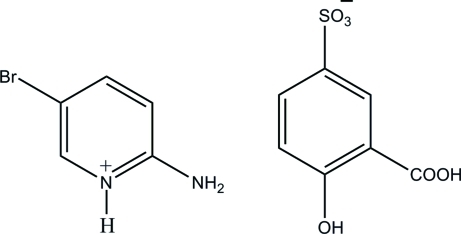


## Experimental

### 

#### Crystal data

C_5_H_6_BrN_2_^+^·C_7_H_5_O_6_S^−^
*M* *_r_* = 391.20
Triclinic, 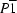

*a* = 7.8425 (2) Å
*b* = 10.8682 (3) Å
*c* = 16.5457 (5) Å
α = 85.207 (2)°
β = 83.290 (2)°
γ = 86.537 (2)°
*V* = 1393.87 (7) Å^3^
*Z* = 4
Mo *K*α radiation
μ = 3.13 mm^−1^
*T* = 100 K
0.26 × 0.14 × 0.09 mm

#### Data collection

Bruker SMART APEXII CCD area-detector diffractometer
Absorption correction: multi-scan (*SADABS*; Bruker, 2009 ▶) *T*_min_ = 0.498, *T*_max_ = 0.772
34040 measured reflections
9322 independent reflections
7366 reflections with *I* > 2σ(*I*)
*R*_int_ = 0.040

#### Refinement

*R*[*F* ^2^ > 2σ(*F* ^2^)] = 0.060
*wR*(*F* ^2^) = 0.181
*S* = 1.11
9322 reflections
397 parameters
H-atom parameters constrained
Δρ_max_ = 1.65 e Å^−3^
Δρ_min_ = −0.83 e Å^−3^

### 

Data collection: *APEX2* (Bruker, 2009 ▶); cell refinement: *SAINT* (Bruker, 2009 ▶); data reduction: *SAINT*; program(s) used to solve structure: *SHELXTL* (Sheldrick, 2008 ▶); program(s) used to refine structure: *SHELXTL*; molecular graphics: *SHELXTL*; software used to prepare material for publication: *SHELXTL* and *PLATON* (Spek, 2009 ▶).

## Supplementary Material

Crystal structure: contains datablocks global, I. DOI: 10.1107/S1600536810033908/ci5165sup1.cif

Structure factors: contains datablocks I. DOI: 10.1107/S1600536810033908/ci5165Isup2.hkl

Additional supplementary materials:  crystallographic information; 3D view; checkCIF report

## Figures and Tables

**Table 1:** Hydrogen-bond geometry (Å, °)

*D*—H⋯*A*	*D*—H	H⋯*A*	*D*⋯*A*	*D*—H⋯*A*
N1*A*—H1*NA*⋯O4*B*	0.99	1.86	2.811 (6)	160
N2*A*—H2*AB*⋯O6*B*^i^	0.90	2.23	3.113 (6)	166
N2*A*—H2*NA*⋯O5*B*	0.80	2.22	2.919 (6)	146
N2*A*—H2*NA*⋯O2*A*^i^	0.80	2.26	2.807 (6)	126
O1*A*—H1*OA*⋯O2*A*	0.82	1.82	2.596 (5)	158
O3*A*—H2*OA*⋯O4*B*	0.90	2.60	3.250 (5)	130
O3*A*—H2*OA*⋯O6*B*	0.90	1.77	2.649 (5)	165
N1*B*—H1*NB*⋯O6*A*^ii^	0.84	2.13	2.859 (6)	145
N2*B*—H2*NB*⋯O5*A*^ii^	0.83	2.34	3.006 (5)	138
N2*B*—H3*NB*⋯O4*A*	0.76	2.27	3.024 (6)	175
O1*B*—H1*OB*⋯O2*B*	0.81	1.89	2.582 (5)	143
O1*B*—H1*OB*⋯O5*A*^iii^	0.81	2.42	3.023 (5)	131
O3*B*—H2*OB*⋯O4*A*^iv^	0.89	1.79	2.661 (5)	166
C5*A*—H5*AA*⋯O1*A*^v^	0.93	2.56	3.188 (6)	125
C10*A*—H10*A*⋯O1*B*^iii^	0.93	2.56	3.406 (6)	151
C10*B*—H10*B*⋯O1*A*^i^	0.93	2.53	3.364 (6)	150

Symmetry codes: (i) 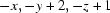; (ii) 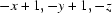; (iii) 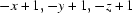; (iv) 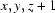; (v) 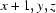.

**Fig. 1.**
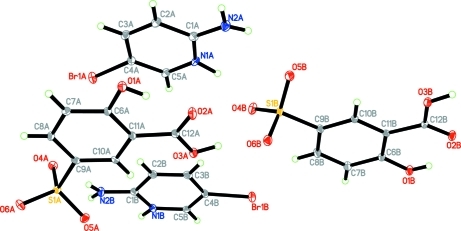
The asymmetric unit of the title compound. Displacement ellipsoids are drawn at the 50% probability level.

**Fig. 2.**
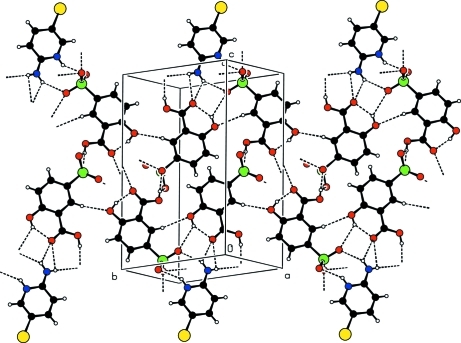
Hydrogen bonding patterns in compound (I).

**Fig. 3.**
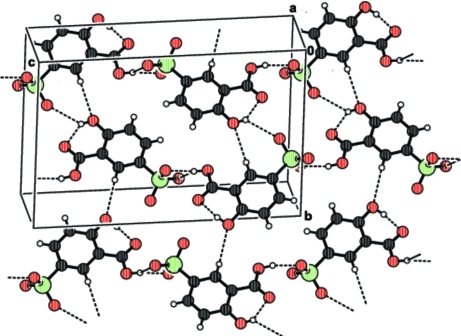
A view of hydrogen-bonded sheet made up of 3-carboxy-4-hydroxy benzenesulfonate anions.

## supplementary crystallographic information

### Comment

Pyridinium derivatives often exhibit antibacterial and antifungal activities (Akkurt *et al.*, 2005). There are numerous examples of 2-amino-substituted pyridine compounds in which the 2-aminopyridines act as neutral ligands (Navarro Ranninger *et al.*, 1985; Krizanovic *et al.*, 1993; Luque *et al.*, 1997; Qin *et al.*, 1999; Yip *et al.*, 1999; Lah *et al.*, 2002; Ren *et al.*, 2002; Rivas *et al.*, 2003) or as protonated cations (Luque *et al.*, 1997; Jin *et al.*, 2000; Albrecht *et al.*, 2003).  We have been interested in hydrogen-bonded systems formed by 2-aminopyridines and carboxylic acids that generate molecular assemblies (Hemamalini & Fun, 2010; Quah *et al.*, 2010). In continuation of our studies of pyridinium derivatives, the crystal structure determination of the title compound has been undertaken.

The asymmetric unit of the title compound consists of two crystallographically independent 2-amino-5-bromopyridinium cations (A and B) and two 3-carboxy-4-hydroxybenzenesulfonate anions (A and B) (Fig. 1). Each 2-amino-5-bromopyridinium cation is planar, with a maximum deviation of 0.015 (1) Å for atom Br1A in cation A and 0.031 (1) Å for Br1B atom in cation B. In the cations, protonation at atoms N1A and N1B lead to a slight increase in the C1A—N1A—C5A [123.9 (4)°] and C1B—N1B—C5B [123.8 (4)°] angles.

In the crystal structure (Fig. 2), the sulfonate group of each 3-carboxy-4-hydroxybenzenesulfonate anion interacts with the corresponding 2-amino-5-bromopyridinium cations via a pair of N—H···O hydrogen bonds forming an *R*_2_^2^(8) ring motif (Bernstein *et al.*, 1995). Here, sulfonate groups mimic the role of the carboxylate groups.  Furthermore, the ionic units are linked by N—H···O, O—H···O and C—H···O (Table 1) hydrogen bonds generating a three-dimensional network. The 3-carboxy-4-hydroxybenzenesulfonate anions self-assemble via O—H···O and C—H···O interactions, leading to the formation of a sheet-like structure, as shown in Fig. 3. There are intramolecular hydrogen bonds between the -OH and -COOH groups in sulfosalicylate anions, which generate *S*(6) ring motifs.

### Experimental

A hot methanol solution (20 ml) of 2-amino-5-bromopyridine (46 mg, Aldrich) and sulfosalicylic acid (54 mg, Merck) were mixed and warmed over a heating magnetic stirrer hotplate for a few minutes. The resulting solution was allowed to cool slowly at room temperature and yellow coloured crystals of the title compound appeared after a few days.

### Refinement

The N- and O-bound H atoms were initially located in a difference map and later allowed to ride on the parent atoms [N–H = 0.76–0.98 Å and O–H = 0.82–0.90 Å], with *U*_iso_(H) = 1.2*U*_eq_(N) and 1.5*U*_eq_(O). C-bound H atoms were positioned geometrically [C–H = 0.93 Å] and refined using a riding model, with *U*_iso_(H) = 1.2*U*_eq_(C). In the final difference Fourier map the highest peak is 0.88 Å from atom Br1A and the deepest hole is 1.55 Å from atom C6A.

### Crystal data

**Table:**

C_5_H_6_BrN_2_^+^·C_7_H_5_O_6_S^−^	*Z* = 4
*M**_r_* = 391.20	*F*(000) = 784
Triclinic, *P*1	*D*_x_ = 1.864 Mg m^−^^3^
Hall symbol: -P 1	Mo *K*α radiation, λ = 0.71073 Å
*a* = 7.8425 (2) Å	Cell parameters from 9916 reflections
*b* = 10.8682 (3) Å	θ = 3.0–31.4°
*c* = 16.5457 (5) Å	µ = 3.13 mm^−^^1^
α = 85.207 (2)°	*T* = 100 K
β = 83.290 (2)°	Plate, yellow
γ = 86.537 (2)°	0.26 × 0.14 × 0.09 mm
*V* = 1393.87 (7) Å^3^	

### Data collection

**Table:**

Bruker SMART APEXII CCD area-detector diffractometer	9322 independent reflections
Radiation source: fine-focus sealed tube	7366 reflections with *I* > 2σ(*I*)
graphite	*R*_int_ = 0.040
φ and ω scans	θ_max_ = 31.7°, θ_min_ = 1.9°
Absorption correction: multi-scan (*SADABS*; Bruker, 2009)	*h* = −11→11
*T*_min_ = 0.498, *T*_max_ = 0.772	*k* = −15→16
34040 measured reflections	*l* = −24→24

### Refinement

**Table:**

Refinement on *F*^2^	Primary atom site location: structure-invariant direct methods
Least-squares matrix: full	Secondary atom site location: difference Fourier map
*R*[*F*^2^ > 2σ(*F*^2^)] = 0.060	Hydrogen site location: inferred from neighbouring sites
*wR*(*F*^2^) = 0.181	H-atom parameters constrained
*S* = 1.11	*w* = 1/[σ^2^(*F*_o_^2^) + (0.0451*P*)^2^ + 14.502*P*] where *P* = (*F*_o_^2^ + 2*F*_c_^2^)/3
9322 reflections	(Δ/σ)_max_ = 0.001
397 parameters	Δρ_max_ = 1.65 e Å^−^^3^
0 restraints	Δρ_min_ = −0.83 e Å^−^^3^

### Special details

**Table:**

Experimental. The crystal was placed in the cold stream of an Oxford Cryosystems Cobra open-flow nitrogen cryostat (Cosier & Glazer, 1986) operating at 100.0 (1) K.
Geometry. All esds (except the esd in the dihedral angle between two l.s. planes) are estimated using the full covariance matrix. The cell esds are taken into account individually in the estimation of esds in distances, angles and torsion angles; correlations between esds in cell parameters are only used when they are defined by crystal symmetry. An approximate (isotropic) treatment of cell esds is used for estimating esds involving l.s. planes.
Refinement. Refinement of F^2^ against ALL reflections. The weighted R-factor wR and goodness of fit S are based on F^2^, conventional R-factors R are based on F, with F set to zero for negative F^2^. The threshold expression of F^2^ > 2sigma(F^2^) is used only for calculating R-factors(gt) etc. and is not relevant to the choice of reflections for refinement. R-factors based on F^2^ are statistically about twice as large as those based on F, and R- factors based on ALL data will be even larger.

### Fractional atomic coordinates and isotropic or equivalent isotropic displacement parameters (Å^2^)

**Table:**

	*x*	*y*	*z*	*U*_iso_*/*U*_eq_	
Br1A	0.34842 (7)	1.03576 (5)	0.09171 (3)	0.02290 (13)	
N1A	0.3053 (5)	1.0274 (4)	0.3409 (2)	0.0164 (7)	
H1NA	0.3775	0.9756	0.3767	0.020*	
N2A	0.1288 (6)	1.1186 (4)	0.4427 (3)	0.0220 (9)	
H2AB	0.0369	1.1714	0.4514	0.026*	
H2NA	0.1650	1.0910	0.4843	0.026*	
C1A	0.1717 (6)	1.1047 (5)	0.3644 (3)	0.0179 (9)	
C2A	0.0822 (6)	1.1683 (5)	0.3013 (3)	0.0181 (9)	
H2AA	−0.0107	1.2229	0.3149	0.022*	
C3A	0.1326 (6)	1.1490 (5)	0.2214 (3)	0.0180 (9)	
H3AA	0.0732	1.1895	0.1806	0.022*	
C4A	0.2754 (6)	1.0674 (5)	0.2008 (3)	0.0161 (8)	
C5A	0.3601 (6)	1.0076 (5)	0.2613 (3)	0.0163 (8)	
H5AA	0.4544	0.9538	0.2485	0.020*	
S1A	0.15205 (14)	0.68598 (11)	0.03315 (6)	0.0126 (2)	
O1A	−0.2400 (5)	0.9811 (3)	0.2821 (2)	0.0180 (7)	
H1OA	−0.2078	0.9424	0.3229	0.027*	
O2A	−0.0584 (5)	0.8634 (4)	0.3883 (2)	0.0227 (8)	
O3A	0.1606 (5)	0.7338 (3)	0.3455 (2)	0.0178 (7)	
H2OA	0.1921	0.7418	0.3952	0.027*	
O4A	0.2922 (4)	0.7619 (3)	−0.0065 (2)	0.0169 (7)	
O5A	0.2150 (5)	0.5735 (3)	0.0766 (2)	0.0196 (7)	
O6A	0.0315 (5)	0.6618 (4)	−0.0241 (2)	0.0194 (7)	
C6A	−0.1432 (6)	0.9151 (4)	0.2271 (3)	0.0134 (8)	
C7A	−0.1724 (6)	0.9374 (4)	0.1452 (3)	0.0162 (8)	
H7AA	−0.2528	0.9992	0.1304	0.019*	
C8A	−0.0827 (6)	0.8683 (4)	0.0861 (3)	0.0150 (8)	
H8AA	−0.1028	0.8838	0.0318	0.018*	
C9A	0.0377 (6)	0.7754 (4)	0.1079 (3)	0.0134 (8)	
C10A	0.0740 (6)	0.7554 (4)	0.1881 (3)	0.0130 (8)	
H10A	0.1576	0.6954	0.2018	0.016*	
C11A	−0.0146 (6)	0.8250 (4)	0.2484 (3)	0.0129 (8)	
C12A	0.0249 (6)	0.8082 (4)	0.3339 (3)	0.0154 (8)	
Br1B	0.82103 (7)	0.51159 (5)	0.36511 (3)	0.02183 (12)	
N1B	0.7997 (5)	0.5274 (4)	0.1191 (2)	0.0151 (7)	
H1NB	0.8554	0.5000	0.0772	0.018*	
N2B	0.6286 (6)	0.6276 (4)	0.0271 (2)	0.0185 (8)	
H2NB	0.6942	0.6071	−0.0134	0.022*	
H3NB	0.5478	0.6641	0.0167	0.022*	
C1B	0.6675 (6)	0.6070 (4)	0.1026 (3)	0.0148 (8)	
C2B	0.5735 (6)	0.6639 (5)	0.1700 (3)	0.0181 (9)	
H2BA	0.4806	0.7187	0.1612	0.022*	
C3B	0.6179 (7)	0.6392 (5)	0.2468 (3)	0.0190 (9)	
H3BA	0.5569	0.6775	0.2903	0.023*	
C4B	0.7588 (6)	0.5541 (5)	0.2600 (3)	0.0157 (8)	
C5B	0.8482 (6)	0.5005 (4)	0.1952 (3)	0.0151 (8)	
H5BA	0.9418	0.4459	0.2030	0.018*	
S1B	0.34815 (15)	0.81374 (11)	0.51288 (7)	0.0149 (2)	
O1B	0.7530 (5)	0.5267 (3)	0.7495 (2)	0.0182 (7)	
H1OB	0.7144	0.5349	0.7967	0.027*	
O2B	0.5619 (5)	0.6350 (4)	0.8632 (2)	0.0196 (7)	
O3B	0.3442 (5)	0.7652 (3)	0.8315 (2)	0.0176 (7)	
H2OB	0.3280	0.7778	0.8844	0.026*	
O4B	0.4611 (5)	0.8372 (4)	0.4372 (2)	0.0239 (8)	
O5B	0.2794 (6)	0.9255 (4)	0.5500 (2)	0.0269 (9)	
O6B	0.2102 (5)	0.7328 (4)	0.5012 (2)	0.0201 (7)	
C6B	0.6577 (6)	0.5938 (4)	0.6973 (3)	0.0133 (8)	
C7B	0.6953 (7)	0.5781 (5)	0.6139 (3)	0.0183 (9)	
H7BA	0.7826	0.5215	0.5963	0.022*	
C8B	0.6037 (6)	0.6462 (5)	0.5575 (3)	0.0186 (9)	
H8BA	0.6293	0.6353	0.5021	0.022*	
C9B	0.4723 (6)	0.7315 (4)	0.5838 (3)	0.0144 (8)	
C10B	0.4318 (6)	0.7471 (4)	0.6658 (3)	0.0131 (8)	
H10B	0.3430	0.8031	0.6828	0.016*	
C11B	0.5235 (6)	0.6792 (4)	0.7235 (3)	0.0121 (7)	
C12B	0.4794 (6)	0.6907 (4)	0.8116 (3)	0.0140 (8)	

### Atomic displacement parameters (Å^2^)

**Table:**

	*U*^11^	*U*^22^	*U*^33^	*U*^12^	*U*^13^	*U*^23^
Br1A	0.0219 (2)	0.0316 (3)	0.0159 (2)	−0.0003 (2)	−0.00412 (17)	−0.00410 (18)
N1A	0.0165 (18)	0.0151 (19)	0.0166 (17)	0.0003 (15)	−0.0026 (14)	0.0048 (14)
N2A	0.023 (2)	0.025 (2)	0.0170 (19)	0.0012 (17)	0.0003 (16)	0.0000 (16)
C1A	0.018 (2)	0.018 (2)	0.017 (2)	−0.0045 (17)	0.0005 (16)	0.0005 (17)
C2A	0.014 (2)	0.019 (2)	0.021 (2)	0.0019 (17)	−0.0016 (16)	0.0014 (17)
C3A	0.016 (2)	0.018 (2)	0.020 (2)	0.0010 (17)	−0.0052 (17)	0.0013 (17)
C4A	0.015 (2)	0.018 (2)	0.0162 (19)	−0.0050 (17)	−0.0020 (15)	0.0011 (16)
C5A	0.0122 (19)	0.017 (2)	0.019 (2)	0.0010 (16)	−0.0019 (16)	0.0007 (16)
S1A	0.0121 (5)	0.0141 (5)	0.0114 (4)	0.0016 (4)	−0.0007 (3)	−0.0027 (4)
O1A	0.0182 (16)	0.0205 (18)	0.0152 (15)	0.0078 (13)	−0.0030 (12)	−0.0050 (13)
O2A	0.0253 (19)	0.028 (2)	0.0148 (15)	0.0126 (15)	−0.0066 (13)	−0.0063 (14)
O3A	0.0188 (17)	0.0184 (17)	0.0166 (15)	0.0095 (13)	−0.0067 (12)	−0.0042 (12)
O4A	0.0147 (15)	0.0189 (17)	0.0164 (15)	−0.0015 (13)	0.0017 (12)	−0.0024 (12)
O5A	0.0242 (18)	0.0155 (17)	0.0181 (16)	0.0067 (14)	−0.0017 (13)	−0.0009 (12)
O6A	0.0183 (17)	0.0240 (19)	0.0175 (15)	−0.0015 (14)	−0.0032 (13)	−0.0093 (13)
C6A	0.0134 (19)	0.013 (2)	0.0137 (18)	0.0030 (15)	−0.0025 (15)	−0.0029 (15)
C7A	0.019 (2)	0.013 (2)	0.0166 (19)	0.0025 (17)	−0.0040 (16)	−0.0015 (15)
C8A	0.016 (2)	0.016 (2)	0.0127 (18)	0.0030 (16)	−0.0035 (15)	−0.0017 (15)
C9A	0.016 (2)	0.0117 (19)	0.0124 (18)	−0.0016 (15)	0.0025 (15)	−0.0038 (14)
C10A	0.0132 (19)	0.012 (2)	0.0141 (18)	−0.0020 (15)	−0.0015 (15)	−0.0038 (15)
C11A	0.0135 (19)	0.014 (2)	0.0121 (17)	0.0013 (15)	−0.0034 (14)	−0.0021 (14)
C12A	0.018 (2)	0.014 (2)	0.0157 (19)	0.0013 (16)	−0.0043 (16)	−0.0027 (15)
Br1B	0.0238 (3)	0.0286 (3)	0.0131 (2)	−0.0010 (2)	−0.00254 (17)	−0.00132 (17)
N1B	0.0132 (17)	0.019 (2)	0.0130 (16)	−0.0017 (14)	0.0005 (13)	−0.0033 (14)
N2B	0.0172 (19)	0.023 (2)	0.0154 (17)	0.0027 (16)	−0.0034 (14)	−0.0017 (15)
C1B	0.014 (2)	0.015 (2)	0.0155 (19)	−0.0031 (16)	0.0002 (15)	−0.0008 (15)
C2B	0.017 (2)	0.016 (2)	0.022 (2)	0.0019 (17)	−0.0020 (17)	−0.0046 (17)
C3B	0.022 (2)	0.017 (2)	0.017 (2)	−0.0029 (18)	0.0031 (17)	−0.0045 (17)
C4B	0.016 (2)	0.019 (2)	0.0124 (18)	−0.0048 (17)	−0.0014 (15)	−0.0009 (15)
C5B	0.016 (2)	0.012 (2)	0.017 (2)	−0.0002 (16)	−0.0024 (16)	−0.0018 (15)
S1B	0.0165 (5)	0.0162 (5)	0.0123 (4)	0.0028 (4)	−0.0046 (4)	−0.0005 (4)
O1B	0.0190 (17)	0.0208 (18)	0.0139 (14)	0.0071 (13)	−0.0032 (12)	−0.0002 (12)
O2B	0.0237 (18)	0.0205 (18)	0.0136 (15)	0.0073 (14)	−0.0040 (13)	0.0006 (12)
O3B	0.0207 (17)	0.0184 (17)	0.0126 (14)	0.0039 (13)	−0.0008 (12)	0.0001 (12)
O4B	0.0250 (19)	0.029 (2)	0.0166 (16)	0.0023 (16)	−0.0023 (14)	0.0037 (14)
O5B	0.038 (2)	0.0215 (19)	0.0210 (17)	0.0152 (17)	−0.0109 (16)	−0.0052 (14)
O6B	0.0193 (17)	0.0232 (19)	0.0186 (16)	−0.0028 (14)	−0.0076 (13)	0.0011 (13)
C6B	0.0105 (18)	0.015 (2)	0.0144 (18)	0.0015 (15)	−0.0022 (14)	−0.0006 (15)
C7B	0.018 (2)	0.020 (2)	0.016 (2)	0.0066 (18)	−0.0025 (16)	−0.0034 (17)
C8B	0.018 (2)	0.022 (2)	0.015 (2)	0.0070 (18)	−0.0050 (16)	−0.0044 (17)
C9B	0.015 (2)	0.016 (2)	0.0130 (18)	0.0021 (16)	−0.0048 (15)	−0.0020 (15)
C10B	0.0128 (19)	0.013 (2)	0.0142 (18)	0.0003 (15)	−0.0031 (14)	−0.0014 (15)
C11B	0.0129 (19)	0.0116 (19)	0.0121 (17)	0.0011 (15)	−0.0030 (14)	−0.0021 (14)
C12B	0.017 (2)	0.013 (2)	0.0124 (18)	−0.0014 (16)	−0.0020 (15)	0.0005 (14)

### Geometric parameters (Å, °)

**Table:**

Br1A—C4A	1.883 (5)	Br1B—C4B	1.874 (5)
N1A—C1A	1.344 (7)	N1B—C1B	1.348 (6)
N1A—C5A	1.367 (6)	N1B—C5B	1.361 (6)
N1A—H1NA	0.98	N1B—H1NB	0.84
N2A—C1A	1.320 (6)	N2B—C1B	1.318 (6)
N2A—H2AB	0.90	N2B—H2NB	0.83
N2A—H2NA	0.80	N2B—H3NB	0.76
C1A—C2A	1.433 (7)	C1B—C2B	1.427 (6)
C2A—C3A	1.365 (7)	C2B—C3B	1.358 (7)
C2A—H2AA	0.93	C2B—H2BA	0.93
C3A—C4A	1.413 (7)	C3B—C4B	1.422 (7)
C3A—H3AA	0.93	C3B—H3BA	0.93
C4A—C5A	1.364 (7)	C4B—C5B	1.364 (6)
C5A—H5AA	0.93	C5B—H5BA	0.93
S1A—O5A	1.455 (4)	S1B—O5B	1.453 (4)
S1A—O6A	1.464 (4)	S1B—O4B	1.461 (4)
S1A—O4A	1.473 (4)	S1B—O6B	1.475 (4)
S1A—C9A	1.765 (4)	S1B—C9B	1.764 (5)
O1A—C6A	1.343 (5)	O1B—C6B	1.347 (6)
O1A—H1OA	0.82	O1B—H1OB	0.82
O2A—C12A	1.225 (6)	O2B—C12B	1.230 (6)
O3A—C12A	1.319 (6)	O3B—C12B	1.322 (6)
O3A—H2OA	0.90	O3B—H2OB	0.89
C6A—C7A	1.399 (6)	C6B—C7B	1.400 (6)
C6A—C11A	1.417 (6)	C6B—C11B	1.414 (6)
C7A—C8A	1.384 (6)	C7B—C8B	1.384 (7)
C7A—H7AA	0.93	C7B—H7BA	0.93
C8A—C9A	1.395 (7)	C8B—C9B	1.399 (7)
C8A—H8AA	0.93	C8B—H8BA	0.93
C9A—C10A	1.386 (6)	C9B—C10B	1.379 (6)
C10A—C11A	1.397 (6)	C10B—C11B	1.397 (6)
C10A—H10A	0.93	C10B—H10B	0.93
C11A—C12A	1.478 (6)	C11B—C12B	1.472 (6)
			
C1A—N1A—C5A	123.9 (4)	C1B—N1B—C5B	123.8 (4)
C1A—N1A—H1NA	126.9	C1B—N1B—H1NB	113.6
C5A—N1A—H1NA	109.2	C5B—N1B—H1NB	122.5
C1A—N2A—H2AB	112.1	C1B—N2B—H2NB	123.2
C1A—N2A—H2NA	135.2	C1B—N2B—H3NB	122.4
H2AB—N2A—H2NA	112.7	H2NB—N2B—H3NB	114.2
N2A—C1A—N1A	119.7 (5)	N2B—C1B—N1B	119.9 (4)
N2A—C1A—C2A	123.3 (5)	N2B—C1B—C2B	123.1 (5)
N1A—C1A—C2A	117.1 (4)	N1B—C1B—C2B	117.0 (4)
C3A—C2A—C1A	120.3 (5)	C3B—C2B—C1B	121.0 (5)
C3A—C2A—H2AA	119.9	C3B—C2B—H2BA	119.5
C1A—C2A—H2AA	119.9	C1B—C2B—H2BA	119.5
C2A—C3A—C4A	119.8 (5)	C2B—C3B—C4B	119.2 (4)
C2A—C3A—H3AA	120.1	C2B—C3B—H3BA	120.4
C4A—C3A—H3AA	120.1	C4B—C3B—H3BA	120.4
C5A—C4A—C3A	119.5 (4)	C5B—C4B—C3B	119.4 (4)
C5A—C4A—Br1A	118.9 (4)	C5B—C4B—Br1B	119.3 (4)
C3A—C4A—Br1A	121.5 (4)	C3B—C4B—Br1B	121.3 (3)
C4A—C5A—N1A	119.5 (5)	N1B—C5B—C4B	119.7 (5)
C4A—C5A—H5AA	120.3	N1B—C5B—H5BA	120.2
N1A—C5A—H5AA	120.3	C4B—C5B—H5BA	120.2
O5A—S1A—O6A	112.6 (2)	O5B—S1B—O4B	113.7 (3)
O5A—S1A—O4A	112.6 (2)	O5B—S1B—O6B	111.5 (3)
O6A—S1A—O4A	111.7 (2)	O4B—S1B—O6B	111.1 (2)
O5A—S1A—C9A	106.2 (2)	O5B—S1B—C9B	106.3 (2)
O6A—S1A—C9A	107.2 (2)	O4B—S1B—C9B	107.4 (2)
O4A—S1A—C9A	106.0 (2)	O6B—S1B—C9B	106.3 (2)
C6A—O1A—H1OA	96.5	C6B—O1B—H1OB	111.2
C12A—O3A—H2OA	109.7	C12B—O3B—H2OB	113.5
O1A—C6A—C7A	117.7 (4)	O1B—C6B—C7B	118.0 (4)
O1A—C6A—C11A	123.0 (4)	O1B—C6B—C11B	122.7 (4)
C7A—C6A—C11A	119.3 (4)	C7B—C6B—C11B	119.3 (4)
C8A—C7A—C6A	120.5 (4)	C8B—C7B—C6B	120.5 (4)
C8A—C7A—H7AA	119.7	C8B—C7B—H7BA	119.7
C6A—C7A—H7AA	119.7	C6B—C7B—H7BA	119.7
C7A—C8A—C9A	120.0 (4)	C7B—C8B—C9B	119.9 (4)
C7A—C8A—H8AA	120.0	C7B—C8B—H8BA	120.1
C9A—C8A—H8AA	120.0	C9B—C8B—H8BA	120.1
C10A—C9A—C8A	120.4 (4)	C10B—C9B—C8B	120.5 (4)
C10A—C9A—S1A	119.1 (4)	C10B—C9B—S1B	119.2 (4)
C8A—C9A—S1A	120.5 (3)	C8B—C9B—S1B	120.2 (3)
C9A—C10A—C11A	120.2 (4)	C9B—C10B—C11B	120.3 (4)
C9A—C10A—H10A	119.9	C9B—C10B—H10B	119.9
C11A—C10A—H10A	119.9	C11B—C10B—H10B	119.9
C10A—C11A—C6A	119.5 (4)	C10B—C11B—C6B	119.6 (4)
C10A—C11A—C12A	121.4 (4)	C10B—C11B—C12B	121.5 (4)
C6A—C11A—C12A	119.1 (4)	C6B—C11B—C12B	118.9 (4)
O2A—C12A—O3A	123.4 (4)	O2B—C12B—O3B	122.1 (4)
O2A—C12A—C11A	122.1 (4)	O2B—C12B—C11B	122.6 (4)
O3A—C12A—C11A	114.4 (4)	O3B—C12B—C11B	115.3 (4)
			
C5A—N1A—C1A—N2A	−179.5 (5)	C5B—N1B—C1B—N2B	−179.8 (4)
C5A—N1A—C1A—C2A	0.6 (7)	C5B—N1B—C1B—C2B	−0.9 (7)
N2A—C1A—C2A—C3A	−179.5 (5)	N2B—C1B—C2B—C3B	179.6 (5)
N1A—C1A—C2A—C3A	0.3 (7)	N1B—C1B—C2B—C3B	0.7 (7)
C1A—C2A—C3A—C4A	−0.9 (7)	C1B—C2B—C3B—C4B	−0.8 (7)
C2A—C3A—C4A—C5A	0.7 (7)	C2B—C3B—C4B—C5B	1.0 (7)
C2A—C3A—C4A—Br1A	179.1 (4)	C2B—C3B—C4B—Br1B	−177.6 (4)
C3A—C4A—C5A—N1A	0.2 (7)	C1B—N1B—C5B—C4B	1.1 (7)
Br1A—C4A—C5A—N1A	−178.3 (3)	C3B—C4B—C5B—N1B	−1.1 (7)
C1A—N1A—C5A—C4A	−0.9 (7)	Br1B—C4B—C5B—N1B	177.5 (3)
O1A—C6A—C7A—C8A	−176.8 (4)	O1B—C6B—C7B—C8B	−178.8 (5)
C11A—C6A—C7A—C8A	3.1 (7)	C11B—C6B—C7B—C8B	0.6 (7)
C6A—C7A—C8A—C9A	0.1 (7)	C6B—C7B—C8B—C9B	0.0 (8)
C7A—C8A—C9A—C10A	−2.9 (7)	C7B—C8B—C9B—C10B	−0.9 (8)
C7A—C8A—C9A—S1A	179.6 (4)	C7B—C8B—C9B—S1B	−177.1 (4)
O5A—S1A—C9A—C10A	22.9 (4)	O5B—S1B—C9B—C10B	27.2 (5)
O6A—S1A—C9A—C10A	143.5 (4)	O4B—S1B—C9B—C10B	149.2 (4)
O4A—S1A—C9A—C10A	−97.1 (4)	O6B—S1B—C9B—C10B	−91.7 (4)
O5A—S1A—C9A—C8A	−159.7 (4)	O5B—S1B—C9B—C8B	−156.5 (4)
O6A—S1A—C9A—C8A	−39.1 (4)	O4B—S1B—C9B—C8B	−34.5 (5)
O4A—S1A—C9A—C8A	80.4 (4)	O6B—S1B—C9B—C8B	84.6 (4)
C8A—C9A—C10A—C11A	2.5 (7)	C8B—C9B—C10B—C11B	1.0 (7)
S1A—C9A—C10A—C11A	179.9 (3)	S1B—C9B—C10B—C11B	177.3 (3)
C9A—C10A—C11A—C6A	0.8 (7)	C9B—C10B—C11B—C6B	−0.4 (7)
C9A—C10A—C11A—C12A	−178.2 (4)	C9B—C10B—C11B—C12B	−177.9 (4)
O1A—C6A—C11A—C10A	176.4 (4)	O1B—C6B—C11B—C10B	178.9 (4)
C7A—C6A—C11A—C10A	−3.5 (7)	C7B—C6B—C11B—C10B	−0.5 (7)
O1A—C6A—C11A—C12A	−4.6 (7)	O1B—C6B—C11B—C12B	−3.5 (7)
C7A—C6A—C11A—C12A	175.5 (4)	C7B—C6B—C11B—C12B	177.1 (4)
C10A—C11A—C12A—O2A	−175.6 (5)	C10B—C11B—C12B—O2B	−177.5 (5)
C6A—C11A—C12A—O2A	5.4 (7)	C6B—C11B—C12B—O2B	5.0 (7)
C10A—C11A—C12A—O3A	7.2 (7)	C10B—C11B—C12B—O3B	2.6 (6)
C6A—C11A—C12A—O3A	−171.8 (4)	C6B—C11B—C12B—O3B	−175.0 (4)

### Hydrogen-bond geometry (Å, °)

**Table:**

*D*—H···*A*	*D*—H	H···*A*	*D*···*A*	*D*—H···*A*
N1A—H1NA···O4B	0.99	1.86	2.811 (6)	160
N2A—H2AB···O6B^i^	0.90	2.23	3.113 (6)	166
N2A—H2NA···O5B	0.80	2.22	2.919 (6)	146
N2A—H2NA···O2A^i^	0.80	2.26	2.807 (6)	126
O1A—H1OA···O2A	0.82	1.82	2.596 (5)	158
O3A—H2OA···O4B	0.90	2.60	3.250 (5)	130
O3A—H2OA···O6B	0.90	1.77	2.649 (5)	165
N1B—H1NB···O6A^ii^	0.84	2.13	2.859 (6)	145
N2B—H2NB···O5A^ii^	0.83	2.34	3.006 (5)	138
N2B—H3NB···O4A	0.76	2.27	3.024 (6)	175
O1B—H1OB···O2B	0.81	1.89	2.582 (5)	143
O1B—H1OB···O5A^iii^	0.81	2.42	3.023 (5)	131
O3B—H2OB···O4A^iv^	0.89	1.79	2.661 (5)	166
C5A—H5AA···O1A^v^	0.93	2.56	3.188 (6)	125
C10A—H10A···O1B^iii^	0.93	2.56	3.406 (6)	151
C10B—H10B···O1A^i^	0.93	2.53	3.364 (6)	150

Symmetry codes: (i) −*x*, −*y*+2, −*z*+1; (ii) −*x*+1, −*y*+1, −*z*; (iii) −*x*+1, −*y*+1, −*z*+1; (iv) *x*, *y*, *z*+1; (v) *x*+1, *y*, *z*.
